# UK Trainee Cardiothoracic Surgeons’ Perceptions of Public Outcome Reporting in Surgery: A Mixed-Methods Study

**DOI:** 10.7759/cureus.20253

**Published:** 2021-12-07

**Authors:** Prasanna Ganeshan, Milad Baburi

**Affiliations:** 1 Anaesthesia/Intensive Care Medicine, New Cross Hospital, Birmingham, GBR; 2 Stroke, Russells Hall Hospital, Birmingham, GBR

**Keywords:** adult thoracic surgery, healthcare policy and management, surgical outcomes research, congenital cardiothoracic surgery, adult cardiac surgery

## Abstract

Background

Since 2004, the Society for Cardiothoracic Surgery in Great Britain and Ireland has reported outcomes of named surgeons. In 2013, the National Health Service England published outcome data for 10 specialties, including cardiothoracic surgery. Before this, no consistent and major stakeholder feedback had occurred. This is the first study to assess UK trainee cardiothoracic surgeons’ perceptions of public outcome reporting (POR) in surgery.

Methodology

In this study, first, an online survey was sent to all trainee cardiothoracic surgeons (n = 257) in the UK. The survey had a response rate of 17%. Second, 10 semi-structured, one-to-one interviews were conducted with trainee cardiothoracic surgeons who had completed the survey.

Results

The majority of respondents opposed the public release of surgeon-specific mortality data in adult cardiac surgery. It is believed to be associated with several consequences, including risk aversion, ‘gaming’, and detriments to the training and development of surgeons. Despite this, the majority of respondents favoured the POR of alternative outcome measures, including unit mortality, which provides a better indicator for the overall quality of care provided to patients.

Conclusions

Trainee cardiothoracic surgeons accept and approve of POR. However, policymakers should refine the current strategy if they are to receive full support from the future of the specialty.

## Introduction

In recent years, the medical profession has faced mounting pressure for greater transparency and disclosure in healthcare. Its intention is to provide patients with information regarding the performance of their clinicians and the hospitals that provide them with care. Cardiothoracic surgery was the first surgical specialty to come under public scrutiny. A catalyst for this was the Bristol inquiry which followed the high death rates associated with congenital cardiac surgery at the Bristol Royal Infirmary [1]. In the wake of this scandal, the UK government mandated the reporting of surgeon-specific mortality data (SSMD) for all cardiothoracic units across the state. In 2004, the Society for Cardiothoracic Surgery in Great Britain and Ireland (SCTS) published the activity and mortality rates for all consultants performing adult cardiac surgery [2].

Since then, the National Health Service (NHS) has been confronted by further concerns regarding patient care. Following the ‘Francis Report’, the NHS Commissioning Board published guidance, re-emphasising the need for greater transparency in the NHS from both staff and groups responsible for the commissioning of services [3]. The document called for units to publish the ‘activity, clinical quality measures and survival rates from national clinical audits for consultants practising’ across 10 specialties. It aimed to improve surgical care, identify ‘outliers’ with high mortality rates, and facilitate transparency and decision-making for patients.

NHS England’s Consultant Outcomes Publication (COP) includes cardiothoracic surgery, for which data are submitted by the National Institute for Cardiovascular Outcomes Research (NICOR). Yet, public outcome reporting (POR) varies between subspecialties, with each using its own risk-adjustment system. Adult cardiac surgery reports 30-day mortality by SSMD, risk-adjusted by the additional [4] or logistic [5] European System for Cardiac Operative Risk Evaluation (EuroSCORE); adult thoracic surgery reports 30-day mortality by unit [6], risk-adjusted by the Thoracoscore [7]; and congenital cardiothoracic surgery reports 30-day mortality by unit [8], risk-adjusted by the Partial Risk Assessment in Surgery model [9].

The open publication of SSMD has posed numerous challenges. Adult cardiac surgery has observed several of these. First, it is thought to have led to risk-averse behaviour [10]. This arises when a surgeon chooses not to operate on a patient due to the high probability of poor outcomes. Second, it is believed to have prompted ‘gaming’, where the intentional ‘upcoding’ of risk factors may result in a lower than appropriate risk-adjusted mortality [11]. Third, it is assumed to have had an effect on the training and development of cardiothoracic surgeons. Khan et al. found a significant decrease in the proportion and variety of operations performed by trainees following the open publication of SSMD [12]. All three above-mentioned concerns are complex and cannot simply be explained by POR. However, there is a perception that it has significantly contributed to these concerns. Hence, the open publication of SSMD has led to the suggestion of alternative outcome measures. These include long-term mortality, readmission rates, and patient satisfaction [13].

Surprisingly, no major or consistent stakeholder feedback occurred before the introduction of COP. As the future of their specialty, it is imperative that trainees have their say. This is the first study to assess UK trainee cardiothoracic surgeons’ perceptions of POR in surgery.

This article was previously presented as a meeting abstract at the Society for Cardiothoracic Surgery in Great Britain and Ireland's Annual Meeting on the 22nd-24th March 2020.

## Materials and methods

A mixed-methods study design was chosen as it would provide a better understanding of trainees’ perceptions of POR. The following two instruments were used to collect data: (1) A survey with a five-point Likert scale, and (2) a semi-structured, one-to-one interview with open-ended questions. An ‘exploratory sequential design’ was employed; quantitative data collection (survey) was followed by qualitative data collection (interviews) [14]. The qualitative data were then used to interpret and explain the findings of the quantitative data.

Survey design

A modified version of the questionnaire developed by Jarral et al. was used [13] (Appendices, Figures 2-6). This was due to two reasons. First, the researchers’ objectives were comparable to those of this study. Second, the questionnaire had already been piloted and feedback was used to refine its language. The questionnaire was modified to consider a trainee’s level of training (specialty surgical training-8 or post completion of training: grace period) and subspecialty of interest (adult cardiac, adult thoracic, or congenital cardiothoracic).

The questionnaire was uploaded onto an online platform (Online Surveys, Jisc, Bristol). In March 2019, a link to the survey was sent to all trainees (n = 257) via the SCTS. The survey was open for two weeks. Trainees were sent a reminder to complete the survey at two time points; a week after the survey was published and on the day that it closed. Those who participated in the survey were informed that their consent was presumed on completion and anonymity was guaranteed.

Interview design

All interviews followed a semi-structured format. The interviewer (a researcher) was able to establish topic direction, with scope for flexibility; investigate topics important to the trainees that were not previously considered; and adapt the interview questions according to the trainees’ responses. A topic guide was used to direct each interview (Appendices, Figures 7, 8). Probes were included to encourage trainees to provide further information but did not suggest specific answers.

Originally, the researchers had planned to interview trainees from the West and East Midlands. Their convenient accessibility and proximity meant that convenience sampling was used. Two consultant cardiothoracic surgeons, both of whom had links with the Health Services Management Centre at the University of Birmingham, helped to recruit trainees. However, due to the researchers’ initial difficulty in recruiting a sufficient number of trainees, snowball sampling was used to obtain further interviews.

Before each interview, the interviewer ensured that each trainee had completed the online survey; only those who had were recruited. In total, 10 face-to-face and telephone interviews were held between March and April 2019. All interviews were audio-recorded by an encrypted dictaphone, enabling verbatim transcription. The transcripts were then anonymised and sent to each trainee as part of respondent validation.

Each trainee signed a consent form. To preserve anonymity, each trainee was allocated to a numerical ID. A key, matching the numerical ID to the trainees’ names, audio recordings of the interviews, and interview transcripts were stored on an encrypted memory stick. Once the study was completed, all paper records were scanned and destroyed.

Statistical analysis

A statistical analysis of the quantitative data was performed (SPSS version 24.0, IBM Corp., Armonk, NY, USA) to assess if there was a relationship between trainees’ responses and their characteristics. Two statistical tests were performed: Pearson’s chi-square or Fisher’s exact and multivariate binomial logistic regression. The five-point Likert scale was converted into a binomial coefficient. Points one and two on the scale (e.g. ‘Strongly Disagree’ and ‘Disagree’) were recorded as one, while points four and five (e.g. ‘Agree’ and ‘Strongly Agree’) were recorded as zero. The midpoint of the scale (e.g. ‘Neither Agree nor Disagree’) was also recorded as zero; this was felt to be the most conservative treatment for the neutral category.

The statistical analyses of the relationship between trainees’ responses and their characteristics are available from the authors on request.

Qualitative analysis

**Figure 1:**
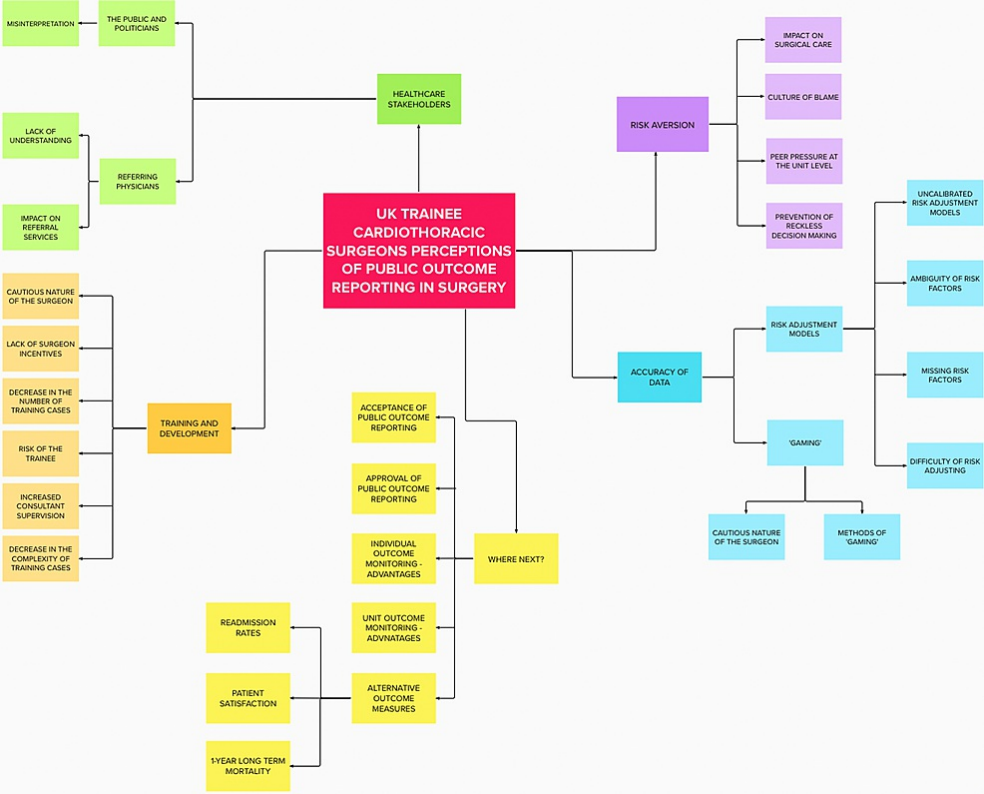
Themes created following the use of Braun and Clarke’s six-step process to qualitative analysis.

**Figure 2:**
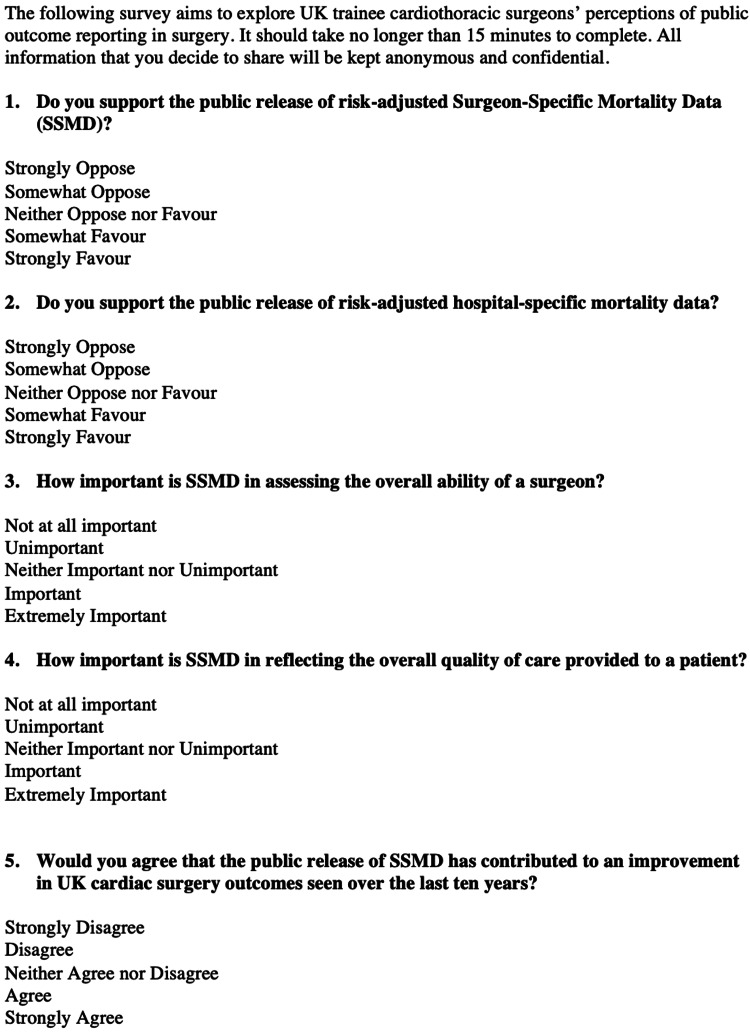
Survey, page 1.

**Figure 3:**
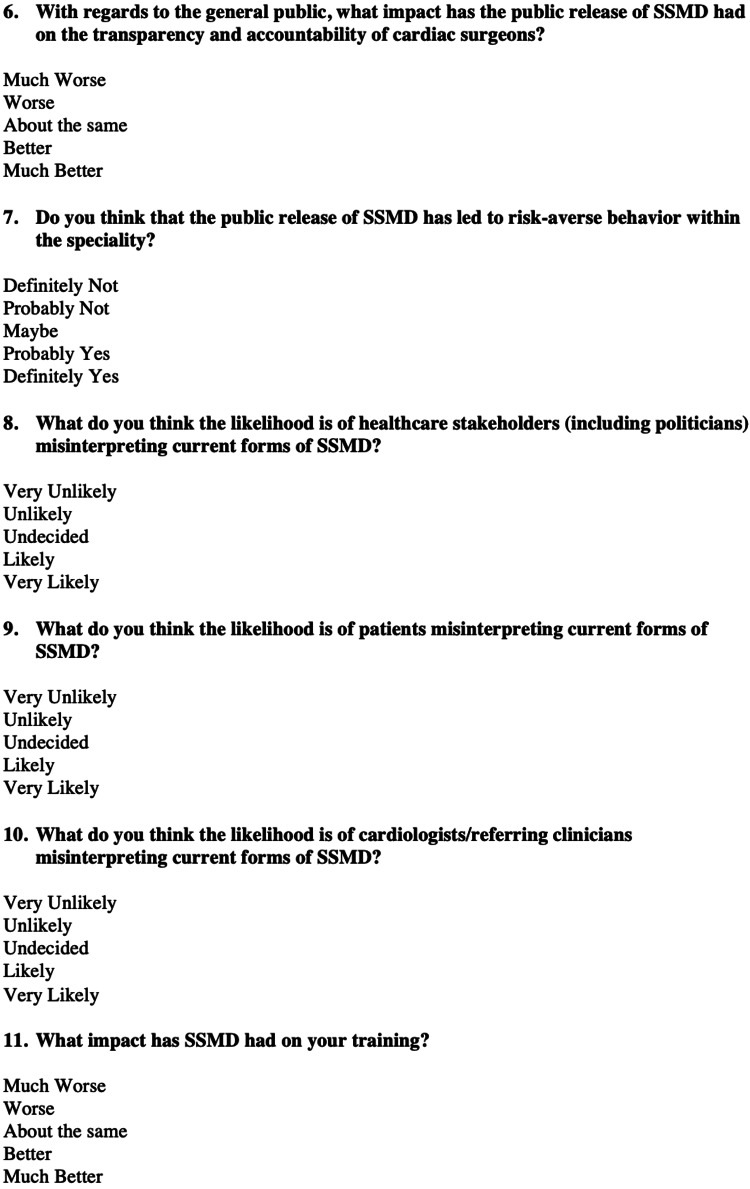
Survey, page 2.

**Figure 4:**
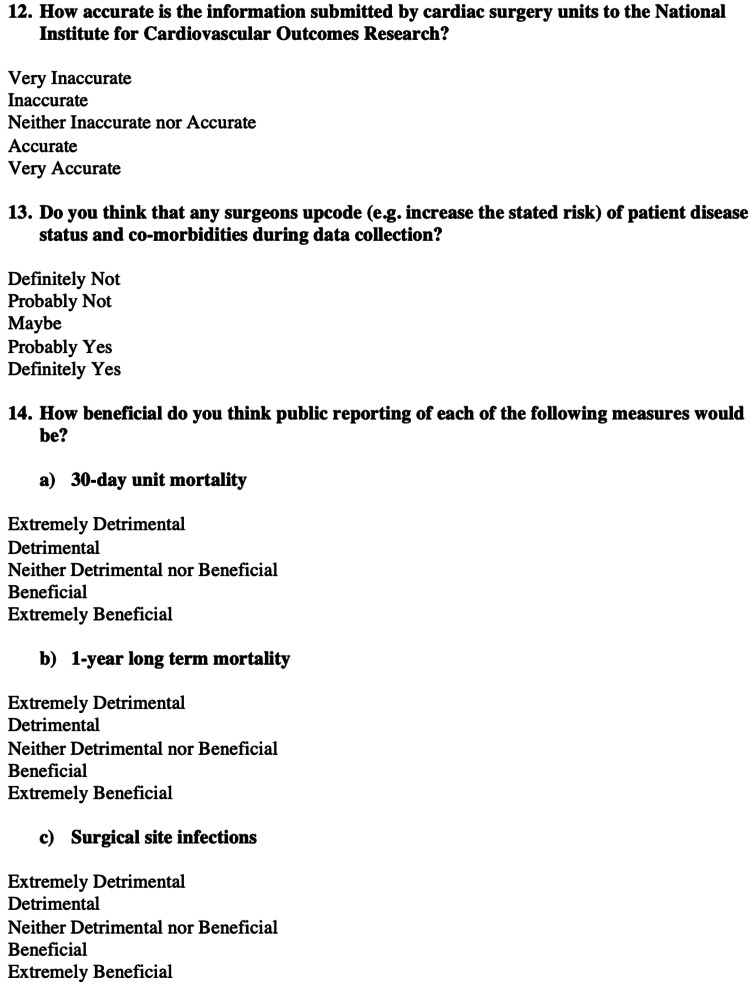
Survey, page 3.

**Figure 5:**
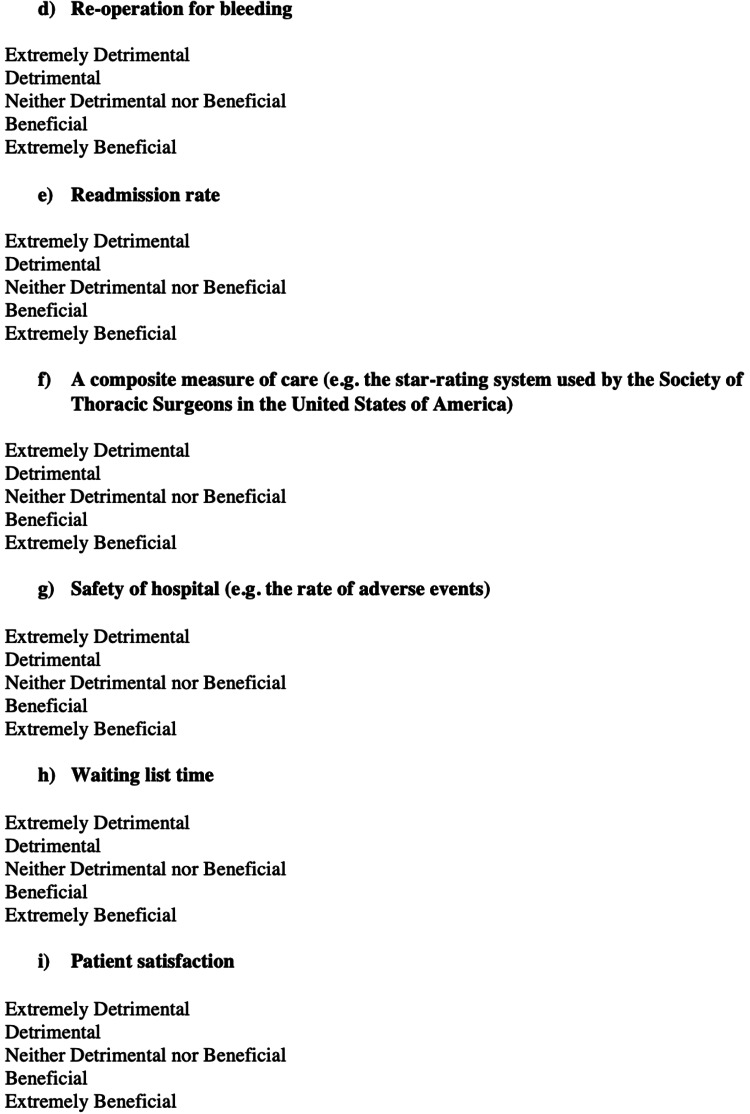
Survey, page 4.

**Figure 6:**
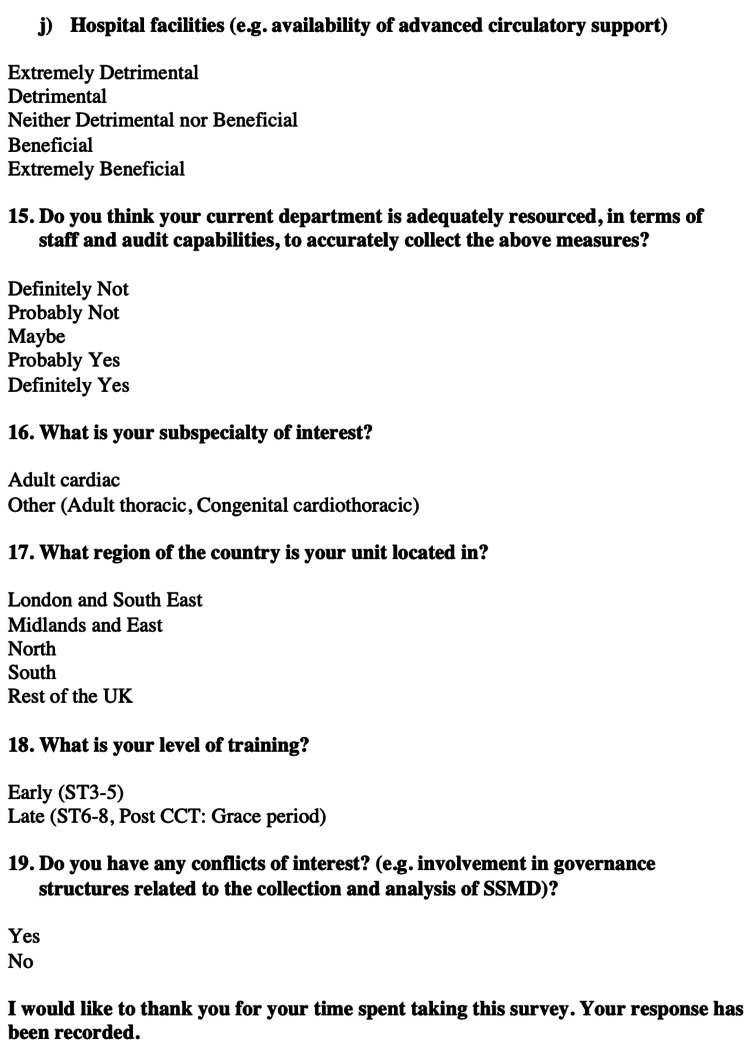
Survey, page 5.

**Figure 7:**
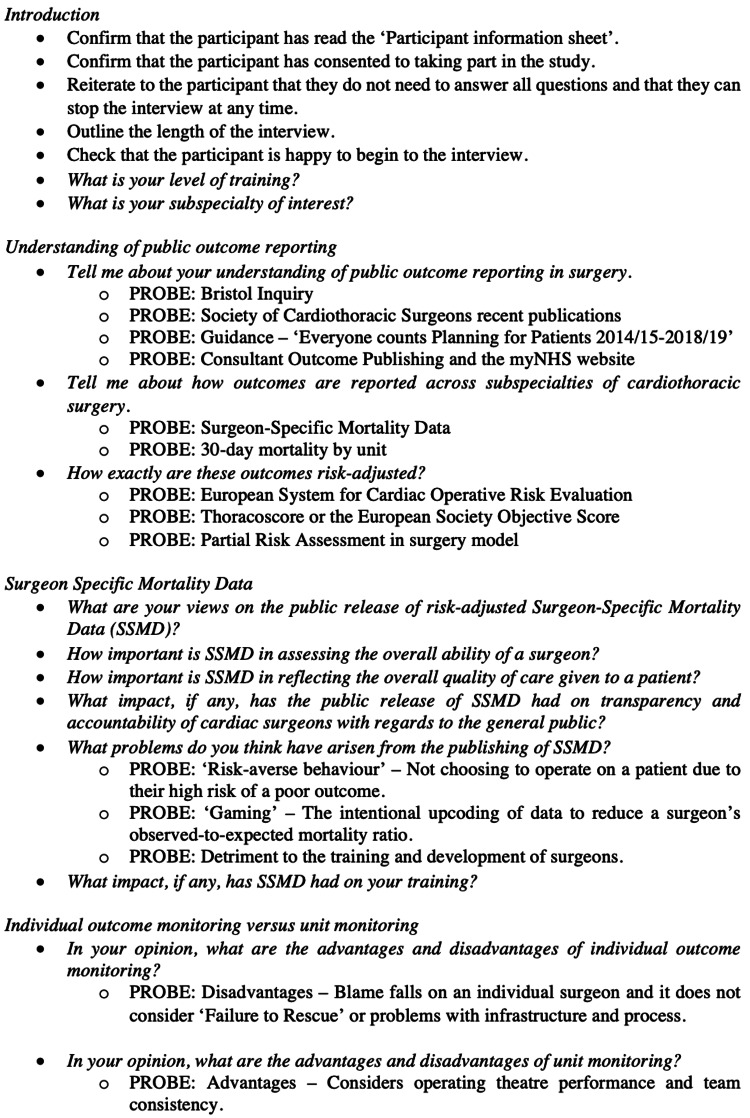
Topic guide used to direct interviews, page 1.

**Figure 8:**
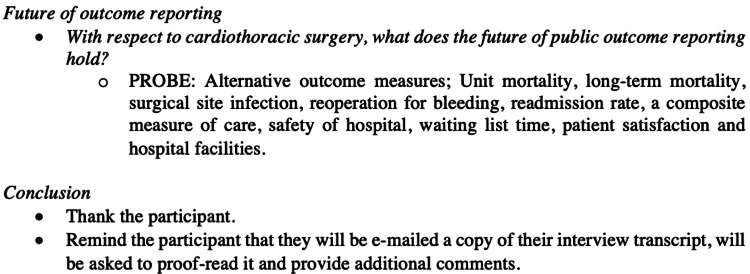
Topic guide used to direct interviews, page 2.

Literature regarding POR and quantitative data obtained from the survey was used to deductively analyse the qualitative data. However, some codes were derived directly and inductively from the raw data. A six-step process, by Braun and Clarke, was used to systematically analyse the data [15] (Figure 1). Due to the subjective nature of coding, interview transcripts were independently coded by two researchers. The two researchers then met to compare their coding and discuss discrepancies.

Data integration

A process for achieving the integration of quantitative and qualitative data in an ‘exploratory sequential design’ is ‘connecting’ [16]. Qualitative data were linked to quantitative data through the sampling frame; only those trainees who had completed the survey were interviewed.

Ethical considerations

This study was authorised at an institutional level (the University of Birmingham, Reference: IREC2018:1323406).

## Results

Survey results 

The overall response rate was 17% (n = 44). Table 1 displays the characteristics of those who responded to the survey. Tables 2, 3 present a full breakdown of trainees’ responses to each question.

**Table 1:** Characteristics of survey respondents. ST: specialty surgical training; CCT: completion of training

Characteristic		Number of responses	%
Level of training	Early (ST3-5)	16	36.4
	Late (ST6-8, post CCT: grace period)	26	59.1
	Unspecified	2	4.5
Geographical location	London and South East	12	27.3
	Midlands and East	13	29.5
	North	14	31.8
	South	2	4.6
	Rest of the UK	3	6.8
Subspecialty of interest	Adult cardiac	30	68.2
	Other (adult thoracic, congenital cardiothoracic)	14	31.8
Involvement in governance structures	Yes	0	0
	No	44	100

**Table 2:** Survey respondents’ opinions on SSMD. SSMD: surgeon-specific mortality data

Question	Number of responses	Answer (%)				
		Strongly Oppose	Somewhat Oppose	Neither Oppose nor Favour	Somewhat Favour	Strongly Favour
Do you support the public release of risk-adjusted SSMD?	44	22.7	31.8	22.7	20.5	2.3
Do you support the public release of risk-adjusted hospital-specific mortality data?	44	4.6	0	6.8	47.7	40.9
		Not at all important	Unimportant	Neither Important nor Unimportant	Important	Extremely Important
How important is SSMD in assessing the overall ability of a surgeon?	44	6.8	20.5	38.6	34.1	0
How important is SSMD in reflecting the overall quality of care provided to a patient?	44	6.8	29.5	27.4	31.8	4.5
		Strongly Disagree	Disagree	Neither Agree nor Disagree	Agree	Strongly Agree
Would you agree that the public release of SSMD has contributed to an improvement in UK cardiac surgery outcomes seen over the last 10 years?	44	6.8	20.5	27.3	40.9	4.5
		Much Worse	Worse	About the same	Better	Much Better
What impact do you think the public release of SSMD had on the transparency and accountability of cardiac surgeons to the general public?	44	4.7	4.7	29.5	54.5	6.6
		Definitely Not	Probably Not	Maybe	Probably Yes	Definitely Yes
Do you think that the public release of SSMD has led to risk-averse behaviour within the specialty?	44	0	2.3	4.5	36.4	56.8
		Very Unlikely	Unlikely	Undecided	Likely	Very Likely
What do you think the likelihood is of healthcare stakeholders (including politicians) misinterpreting current forms of SSMD?	44	0	0	4.5	36.4	59.1
What do you think the likelihood is of patients misinterpreting current forms of SSMD?	44	0	0	6.8	31.8	61.4
What do you think the likelihood is of cardiologists/referring clinicians misinterpreting current forms of SSMD?	44	0	0	20.5	52.2	27.3
		Much Worse	Worse	About the same	Better	Much Better
What impact has SSMD had on your training?	44	18.2	59.1	18.2	4.5	0
		Very Inaccurate	Inaccurate	Neither Inaccurate nor Accurate	Accurate	Very Accurate
How accurate is the information submitted by cardiac surgery units to the National Institute for Cardiovascular Outcomes Research?	43	0	7	30.2	55.8	7
		Definitely Not	Probably Not	Maybe	Probably Yes	Definitely Yes
Do you think any surgeons upcode (e.g. increase the stated risk) patient disease status and co-morbidities during data collection?	44	6.8	18.2	29.6	38.6	6.8

**Table 3:** Survey respondents’ opinions on alternative outcome measures.

Question	Number of responses	Answer (%)				
		Extremely Detrimental	Detrimental	Neither Detrimental nor Beneficial	Beneficial	Extremely Beneficial
How beneficial do you think public reporting of each of the following measures would be?	44					
a) 30-day unit mortality		2.3	11.4	11.4	59.1	15.9
b) 1-year long-term mortality		2.3	9.1	18.1	52.3	18.2
c) Surgical site infection		2.3	4.5	20.5	54.5	18.2
d) Reoperation for bleeding		2.3	15.9	25	43.2	13.6
e) Readmission rate		4.5	4.5	25	52.4	13.6
f) A composite measure of care		7	4.7	25.6	41.9	20.9
g) Safety of hospital (e.g. the rate of adverse events)		2.3	4.5	15.9	50	27.3
h) Waiting list time		2.3	11.6	32.6	34.9	18.6
i) Patient satisfaction		2.3	0	13.6	52.3	31.8
j) Hospital facilities (e.g. the availability of advanced circulatory support)		2.3	6.8	31.8	42.3	15.9
		Definitely Not	Probably Not	Maybe	Probably Yes	Definitely Yes
Do you think your current department is adequately resourced, in terms of staff and audit capabilities to accurately collect the above measures?	44	25	22.7	15.9	34.1	2.3

Interview characteristics

Table 4 displays the characteristics of those who were interviewed.

**Table 4:** Characteristics of interviewees. ST: specialty surgical training; CCT: completion of training

Interview number	Level of training	Region of unit	Subspecialty of interest	Involvement in governance structures
1	ST5	Midlands and East	Adult thoracic	No
2	ST7	Midlands and East	Adult cardiac	No
3	ST6	Midlands and East	Adult thoracic	No
4	ST6	Midlands and East	Adult cardiac	No
5	ST5	Midlands and East	Adult cardiac	No
6	ST8	Midlands and East	Adult thoracic	No
7	ST6	Midlands and East	Congenital cardiothoracic	No
8	ST3	Midlands and East	Congenital cardiothoracic	No
9	Post CCT: grace period	London and South East	Adult cardiac	No
10	ST7	London and South East	Adult cardiac	No

Key findings

Risk Aversion

An overwhelming majority of trainees believed that SSMD has led to risk-averse behaviour (93.2% yes, 2.3% no, and 4.5% maybe). All interviewed trainees stated that risk aversion is rife. Many agreed that it has arisen from a fear that high-risk patients may impact a surgeon’s mortality rate and the unnecessary culture of blame established by SSMD:

“By means of protecting one’s numbers and reputation, one may shy away from taking on a case that, in years gone by, they would’ve.” - Interviwee 6

As one trainee claimed, risk aversion has also been mediated by peer pressure. When one’s peers are risk-averse, an individual within a unit is also likely to adopt this behaviour:

“A surgeon doesn’t want to be the only one accepting the high-risk cases in his unit … the practices of … his colleagues can influence his own actions … taking on a high-risk case can be a sign of overconfidence.” - Interviewee 8

In turn, trainees agreed that surgeons are reluctant to offer surgery to the ‘sickest’ patients or provide them with the ‘gold standard’. Thus, surgeons ‘do not work in ways that best serve their patient’s interests’:

“He [a consultant cardiac surgeon] would never attempt a repair [of the mitral valve] if he thought that there was even a small risk of the patient requiring a second repair or a complete replacement … in a lot of instances the valve was repairable.” - Interiewee 3

On the other hand, not all trainees regarded risk aversion as bad. As two trainees agreed, SSMD has prevented some less experienced surgeons from making reckless decisions due to a fear of repercussions. Thus, risk aversion ensures that high-risk cases are taken on by experienced surgeons.

‘Appropriate and well-attended’ multi-disciplinary team (MDT) meetings are recognised as a potential solution to risk aversion. The discussion of a complex case can facilitate coordinated decision-making that prevents patients from being inappropriately denied an operation. However, as one trainee explained, MDT meetings may not always provide the answer:

“When patients are discussed at an MDT meeting, the surgeon often presents the information in a way that is adequate enough to obtain the decision they wanted.” - Interviewee 4

Accuracy of Data

The majority of trainees believed that the data submitted to the NICOR is accurate (62.8% accurate, 7% inaccurate, and 30.2% neither). While interviews with trainees confirmed this finding, several concerns were raised. All trainees agreed that the current risk-adjustment models are uncalibrated. Both the EuroSCORE, logistic and additive, and the Thoracoscore overpredict the risk of mortality in the highest and lowest risk patients. Furthermore, several trainees agreed that both risk-adjustment models are incomplete; they do not consider some ‘crucial’ factors that would classify a patient as high-risk:

“It [Thoracoscore] doesn’t take into account a lot of factors like ... the individual variables of a patient’s CPET [Cardiopulmonary Exercise Tolerance Testing] ... and the TLCO [Transfer Factor for Carbon Monoxide].” - Interviewee 10

As one trainee noted, inaccuracies in data can also arise because the current EuroSCORE definitions do not accurately reflect the risk of comorbidities in patients undergoing surgery:

“Chronic lung disease ... is defined as the long-term use of steroids or bronchodilators ... some of our patients, with some form of chronic lung disease, do not fall into this definition.” - Interviewee 7

A few trainees also raised concerns over the ambiguity in the definitions of certain EuroSCORE variables. Misinterpretations among surgeons can lead to inconsistencies in the accuracy of data, resulting in variations in the predicted mortality rate among a similar cohort of patients:

“Poor mobility is defined as a severe impairment in mobility due to a musculoskeletal or neurological problem ... but what is severe?” - Interviewee 6

The majority of trainees believed that consultant surgeons upcode patient disease status and co-morbidities during data collection (45.4% yes, 25% not, and 29.6% neither). More often than not, trainees cited ‘gaming’ as ‘legitimate’ but explained that it occurs due to the cautious nature of surgeons rather than their dishonesty. ‘Gaming’ is achieved in several ways. These include more thorough investigations during the pre-operative period and the use of the ‘worst’ available data to ‘improve’ the risk profile of patients:

“Investigations are conducted for all potential comorbidities ... if you were to perform a PFT [Pulmonary Function Test] on every patient ... every now and again you will find someone with a poor lung function.” - Interviewee 7

“If there are several measurements for the pulmonary blood pressure, you could choose to use the highest value.” - Interviewee 9

Training and Development

The majority of trainees believed that training has worsened due to the open publication of SSMD (77.3% worse, 4.5% better, and 18.2% same). Interviews revealed that POR has created a ‘disruptive’ environment for training. Trainees believed that consultants are reluctant to provide them with training opportunities due to a lack of incentives; SSMD does not distinguish between the cases performed by a consultant and their trainees. Consultants are more cautious of the risk that a trainee may pose to a patient due to their lack of experience, and are therefore fearful that this will reflect in their SSMD:

“As well as accepting the risk of the patient, they [consultants] must accept the additional risk that we, as trainees, pose.” - Interviewee 6

All trainees attributed a decline in the number and complexity of cases provided to the POR of SSMD. One trainee voiced their frustration by stating that a lack of experience was preventing them from becoming ‘consultants of the future’. However, as another trainee pointed out, it is crucial to ensure that patients have the best chance of survival. Therefore, the complexity of a case provided to a trainee should be appropriate to their level of training.

All trainees associated the POR of SSMD with an increase in consultant supervision. Some trainees believed that this has been detrimental to their training; it has prevented them from becoming independent surgeons. Despite this, one trainee indicated that increased supervision has been beneficial; consultants provide trainees with invaluable advice on how to improve:

“My surgical ability has developed more when I’ve had a consultant sat opposite me than when I haven’t.” - Interviewee 7

Several trainees also stated that POR has had less of an impact on training in adult thoracic compared to adult cardiac surgery. Consultant thoracic surgeons are perceived as more liberal with their approach to training as unit outcome monitoring often means that they are less accountable to their patients.

Healthcare Stakeholders

An overwhelming proportion of trainees believed that the misinterpretation of SSMD by the public (93.2% likely, 0% unlikely, and 6.8% undecided) and politicians (95.5% likely, 0% unlikely, and 4.5% undecided) is likely. In-depth interviews revealed that all trainees perceived members of both groups to have an ‘over-simplistic’ interpretation of the data due to a lack of understanding of statistical methods. Often, surgeons with a lower risk-adjusted mortality rate are believed to be better.

As one trainee declared, misunderstandings arise from a flaw in terminology. The term SSMD creates a perception that the death of a patient is solely related to a surgeon and their operation, not factors beyond their control. Therefore, alternative terminology should be considered:

“The data that is being used ought to be more accurately described as ‘all-cause mortality of patients who have had an operation by an individual consultant surgeon.’” - Interviewee 1

Trainees believe that the misinterpretation of SSMD has resulted in ‘severe’ and ‘unnecessary’ consequences. These include ‘scapegoating’ and intimidation:

“It is the surgeons that take the hit [for poor outcomes] … in the past, we’ve seen the media falsely accuse surgeons of their seemingly poor patient outcomes.” - Interviewee 2

“I know of a consultant who’d perform mitral valve repairs … he had a few bad days, none of which were his fault … he was informed by senior figures that he could no longer perform mitral valve repairs, or he’d risk gardening leave.” - Interviewee 3

The majority of trainees also believed that referring physicians are likely to misinterpret SSMD (79.6% likely, 0% unlikely, and 20.4% undecided). As in-depth interviews revealed, referring physicians, with their lack of surgical knowledge, are likely to have ‘very little to no understanding of the intricacies of surgery’. It is this lack of understanding that may mean that cardiologists do not discuss SSMD with their patients and that it does not influence their referral practice. However, one trainee disagreed:

“The reporting of surgeon outcomes has … certainly caused a change in referral patterns. Two years ago, hospital A was going through a few problems. As a result, referral centres such as B, C and D were sending their patients to hospital E.” - Interviewee 3

Where Next?

All interviewed trainees accepted and approved of POR. While admitting that it would prove difficult to backtrack on already established practice, trainees believe that improvements can and must be made:

“It's a journey that will only improve with time.” - Interviewee 2

The majority of trainees either strongly or somewhat opposed the public release of SSMD (54.5% oppose, 22.8% favour, and 22.7% neither). However, a significant proportion of trainees favoured the public release of hospital-specific mortality data (88.6% favour, 4.5% oppose, and 6.9% neither).

In-depth interviews revealed a need for adult cardiac surgery to publicly report unit mortality as opposed to SSMD. Trainees stated that the advantages of unit outcome monitoring can be seen in adult thoracic and congenital cardiothoracic surgery. These include reduced risk aversion, greater opportunities for training and development, and increased awareness of team accountability.

Nevertheless, trainees recognised that there are also benefits to individual outcome monitoring, namely, the ability to monitor one’s performance and identify struggling surgeons. However, the latter can promote a ‘name, blame and shame culture’. Therefore, trainees emphasised the importance of SSMD being collected, analysed, and acted on at the level of the unit.

Table 3 presents trainees’ opinions on the public reporting of alternative outcome measures. Although the majority of respondents saw a benefit to the public reporting of such measures, they believed that their current department is not adequately resourced, in terms of staff and audit capabilities, to collect the necessary data (47.7% not, 36.4% yes, and 15.9% maybe).

The majority of trainees believed that the public reporting of one-year long-term mortality is beneficial (70.5% beneficial, 11.4% detrimental, and 18.1% neither). Those at a later stage of their training (ST6-8 and post CCT: grace period) were significantly more in favour of the public reporting of this outcome measure (p = 0.011).

As one trainee noted, one-year long-term mortality provides a better depiction of a patient’s expectations:

“We need to consider what actually matters to a patient … Not one patient is going to undergo a CABG [Coronary Artery Bypass Graft] just to survive for 30 days.” - Interviewee 1

However, as several trainees highlighted, the outcome measure may also falsely attribute the death of a patient to a surgeon:

“During the one year after an operation, a patient may pass away … from a fall or a chest infection. These may be unrelated to the operation … but the patient’s death would still be classified under a surgeon’s outcomes” - Interiewee 3

The majority of trainees believed that the public reporting of readmission rates is beneficial (66% beneficial, 9% detrimental, and 25% neither). Interviews with trainees confirmed this finding. All trainees agreed that a surgeon’s readmission rate is representative of their surgical ability. However, as two trainees pointed out, important considerations must be made. First, a clear time frame for attributing the cause of readmission to surgery must be defined:

“We know that if they’re readmitted within 48 hours it’s a failed discharge … If they’re readmitted beyond a week, it’s not usually due to a surgical mistake.” - Interviewee 10

Second, data capture must be linked to Hospital Episode Statistics to record a patient’s readmission to any unit rather than just the unit of surgery:

“The only concern I have with readmission rate it is the way that it’s measured … readmission rate to the same acute trust … Patients may not … always be readmitted to the same unit that they had their operation.” - Interviewee 1

An overwhelming majority of trainees believed that the public reporting of patient satisfaction is beneficial (84.1% beneficial, 2.3% detrimental, and 13.6% neither). Eight of the ten trainees interviewed agreed with the consensus. As one trainee indicated, surgeons are ‘providers’ and their patients are ‘customers’. Therefore, like any business, an understanding of a consumer’s satisfaction allows for improvements. However, two trainees drew attention to the potential flaws of this outcome measure; patient satisfaction is subjective and may be influenced by factors beyond the surgeon’s control:

“It [patient satisfaction] has many influences … the interaction with staff other than the surgeon ... the noise on the ward, the food.” - Interviewee 10

## Discussion

Risk aversion

The majority of trainees were of the opinion that the public release of SSMD has led to risk-averse behaviour. This is worrying as it implies that a surgeon may disregard the principle of beneficence.

Behavioural science provides an insight into trainees’ perceptions behind the causes of risk-averse behaviour. According to the ‘rational choice theory’, an individual relies on rational calculations to accomplish outcomes that are in line with their personal objectives [17]. Hence, a surgeon may choose to be risk-averse because the marginal utility for reducing loss is far greater than that of increasing gain. Given the current structure of the NHS, this is likely to hold true. As findings from this study reveal, surgeons may not be rewarded for accepting a complex case as they risk scapegoating, suspension or job loss, and intimidation should a patient die.

‘Gaming’

The majority of trainees believe that ‘gaming’ occurs during data collection. Findings from this study indicate that surgeons experience severe levels of stress and are fearful of the repercussions of poor clinical outcomes. A cross-sectional survey of over 10,000 physicians in the UK proposed that those who received complaints or underwent investigations were 77% more likely to suffer from moderate-to-severe depression [18]. Therefore, it is not irrational to suggest that some surgeons may demonstrate atypical behaviour, such as ‘gaming’, as a mechanism of coping with stress [13].

Despite the regional variation, the current process of data entry requires a consultant cardiothoracic surgeon, or a trainee, to submit their case data to an institutional database. Currently, NICOR does not implement any methods for checking the accuracy of inputted data. Concerns about ‘gaming’ provide support to the view that independent non-conflicted parties should perform data entry [13].

Problems with risk-adjustment models

Despite both the additive and logistic EuroSCORE having been previously shown to have a high level of accuracy [19], a meta-analysis demonstrated that the predicted mortality rate is often greater than observed, particularly in high-risk patients [20]. Variations in a risk model’s predicted mortality are due to an increasing prevalence of high-risk patients, believed to be attributed to advancements in diagnostic and interventional cardiology [21]. Therefore, risk-adjustment models from earlier periods cannot be used when the aim of outcome analysis concerns the determination of a trend in mortality over time. Old data fail to consider developments in treatment and changes in case-mix.

Flaws in methodology can also prevent a risk-adjustment model from predicting an individual’s risk. The application of a logistic regression model leads to a survival curve that mathematically demonstrates a multiphasic and complex behaviour [22]. This cannot achieve enough statistical power to attain sufficient accuracy for individual predictions.

Individuals may also provide various interpretations to the categorical risk factors described by both the additive and logistic EuroSCORE. Even with clearly stated definitions, a degree of personal interpretation can occur resulting in a variation in risk scores [23].

Training and development

The majority of trainees believed that the open publication of SSMD has been detrimental to their training and development. In particular, those interviewed complained of a reduction in the number and complexity of cases performed and an increase in consultant supervision. These findings are reflected in the results of a previous study [13].

However, changes in surgical training cannot always be attributed to the impact of SSMD. Alterations in the referral practices of cardiologists and a rise in the use of waiting list initiatives may have caused a reduction in the absolute number of training cases. In addition, the publication of SSMD has coincided with an increase in the risk profile of patients. Therefore, it is likely that consultants are more hesitant to provide independent cases when a greater level of surgical competency is required. This corresponds to Gawande’s ‘learning curve’; there are increased risks associated with being the subject of an invasive procedure when performed by a trainee [24]. Several studies have investigated the relationship between the seniority of a surgeon and clinical outcomes. One study found no difference in outcomes between trainees and consultants who performed colorectal resections [25]. Conversely, another study found long-term outcomes to be poorer in patients undergoing elective total hip replacement when the procedure was performed by trainees rather than consultants [26].

The use of surgeon-specific mortality data by cardiologists

Several interviewed trainees believed that cardiologists do not discuss SSMD with their patients and that it does not influence their referral practice. These findings are confirmed by results of a previous survey from New York, conducted after the introduction of the Cardiac Surgery Reporting System [27].

It is argued that, in an environment where POR exists, cardiologists have an ethical obligation to use the available data to refer clinically appropriate patients to the best available cardiac surgeon [28]. This is in accordance with non-maleficence. Therefore, by not using SSMD, cardiologists may not act in the best interests of their patients.

Limitations

This study has a number of limitations worth noting. The discussion of these limitations is organized according to the method of data collection.

Survey

The survey response rate (17%) was lower than anticipated. However, in view of the politically sensitive nature of the research topic and the trainees’ time constraints, it was not unexpected. Social exchange theory states that an individual will exchange knowledge when they perceive the rewards to be greater than or equal to the costs [29]. Therefore, design and incentive-based approaches can be used to improve response rates. The researchers adopted a few design-based approaches; the survey was brief and trainees were sent reminders to complete the survey on two separate occasions. Monetary incentives may have also increased the response rate to the survey. Unfortunately, the researchers could not provide trainees with compensation for their time due to the financial constraints of this study.

The survey also incorporated a number of leading questions used in the original questionnaire. Such questions may compel respondents to answer in a particular way.

Interviews

The researchers used snowball sampling to recruit two trainees for interviews. While advantageous in gaining niche participants, it increased the risk of selection bias [30]. The initial participants were likely to recommend trainees who they knew well, and it is possible that the trainees shared similar traits and characteristics. Hence, the perceptions of a small subgroup of the study population may have been obtained.

The researchers also attempted to achieve respondent validation. Each interviewed trainee was emailed a copy of their interview transcript to proofread and provide additional comments. Unfortunately, the researchers did not receive any feedback.

## Conclusions

POR is part of an initiative to create greater transparency and accountability within the NHS. It was initially triggered by failures in clinical governance and is tied with political initiatives surrounding patient choice. However, POR in cardiothoracic surgery is not without controversy. Before its introduction, policymakers failed to obtain major and consistent feedback from healthcare stakeholders. This included trainees, the future of their specialty. Therefore, by providing them with a voice, this study aims to bring a fresh outlook on a widely debated topic.

The findings from this study reveal that trainees accept and approve of POR in surgery. However, the open publication of SSMD should be reconsidered for several reasons. Trainees believe that the additional decision-making that it has caused in already stressed surgeons has resulted in a number of far-reaching consequences: risk aversion, ‘gaming’, and detriments to the training and development of surgeons. Although the open publication of SSMD has led to an improvement in patient outcomes, such problems warrant a shift to the POR of unit-based mortality. Furthermore, there is a clear agreement among trainees that policymakers should consider the POR of further complex and multidimensional outcome measures, including long-term mortality, readmission rates, and patient satisfaction.
